# Modeling of Environmental Factors Affecting the Prevalence of Zoonotic and Anthroponotic Cutaneous, and Zoonotic Visceral Leishmaniasis in Foci of Iran: a Remote Sensing and GIS Based Study

**Published:** 2018-03-18

**Authors:** Abdol Ali Golpayegani, Ali Reza Moslem, Amir Ahmad Akhavan, Azam Zeydabadi, Amir Hossein Mahvi, Ahmad Allah-Abadi

**Affiliations:** 1 School of Public Health, Tehran University of Medical Sciences, Tehran, Iran; 2 Bam University of Medical Sciences, Bam, Iran; 3 National Institute of Health Research, Ministry of Health, Tehran, Iran; 4 Sabzevar University of Medical Sciences, Sabzevar, Iran; 5 Institute for Environmental Research, Tehran University of Medical Sciences, Tehran, Iran; 6 Center for Solid Waste Research, Institute for Environmental Research, Tehran University of Medical Sciences, Tehran, Iran; 7 Leishmaniasis Research Center, Sabzevar University of Medical Sciences, Sabzevar, Iran

**Keywords:** Leishmaniasis, GIS, Remote sensing, Environmental variables, Nonlinear regression

## Abstract

**Background:**

Leishmaniasis is a re-emerging serious international public health problem, and both visceral and cutaneous types of leishmaniasis became important endemic diseases in Iran. In this study, the relationships between environmental factors (vegetation and elevation) and the prevalence of diseases have been investigated.

**Methods:**

All international and national online databases were searched by terms such as leishmaniasis, incidence, prevalence and other related words attributed to Iran and published until first quarter of 2015. The developed database in Excel, later imported to the ArcMap for spatial analyst and mapping. Afterwards, the software was used for modeling the relationship between the prevalence/incidence and environmental variables (vegetation and elevation) by both linear and nonlinear regression.

**Results:**

After mapping the prevalence data from 144 studies, considering non-parametric ANOVA, the tendency of zoonotic visceral leishmaniasis to presence in high elevation and high vegetation was more than Anthroponotic and zoonotic cutaneous leishmaniasis. While linear regression showed weaker results for modeling, however, additive nonparametric regression analysis suggested that 10km buffers for elevation, and 10 as well as 50km buffers for vegetation could contribute in better fitness in modeling of these variables.

**Conclusion:**

The detailed maps for distribution of disease concluded. The nonlinear regression is a reliable predictor of the relationship between environmental factors and disease incidence, although more and wide researchers are needed to confirm it.

## Introduction

Leishmaniasis –a re-emerging serious international public health problem- is considered as one of the most neglected tropical diseases. The disease is distributed in new foci due to influence of many risk factors, including environmental factors (1–4). Almost 200000 to 400000 new cases of visceral leishmaniasis (VL), 0.7 million to 1.3 million new cases of cutaneous leishmaniasis (CL) and 20000 to 30000 deaths annually are the estimations of WHO last report about importance of the disease (2).

Two dominant types of leishmaniasis there in Iran are: zoonotic visceral or kala-azar (ZVL), the most serious and fatal type of the disease, and both types of cutaneous leishmaniasis namely urban form or anthroponotic cutaneous leishmaniasis (ACL) and rural form or zoonotic cutaneous leishmaniasis (ZCL). *Leishmania donovani* complex are the causes of ZVL protozoan disease which can lead to patient mortality (5). Evidence indicates an increase in the incidence of disease in the Old and New Worlds at the early years of the present century. In recent decades, ZVL became an important endemic disease in the Northwest (6), South and Southwest (7), and Northeast (8) of Iran, especially in nomads. The majority of ZVL cases (92.8%) in country were found among children with age under 12yr old (9). The most recently, direct agglutination test (DAT) as a simple, affordable and Practical test, has found its way as a special quick and accurate test. Thus it has been recommended and applied widely for clinical diagnosis and seroprevalence studies of ZVL in zoonosis and anthroponotic reservoirs in endemic and nonendemic areas of Iran (10). Due to ecologically dependence of the disease vectors and reservoirs to environment, outbreaks mostly are common in lowlands rural or suburban areas (less than 600M elevation), with a heavy annual rainfall, a moderate temperature (15–38 °C), and almost dense vegetation (2).

Countries of Mediterranean coasts (Algeria and Syrian Arab Republic), South Americas (Brazil and Colombia), and Middle East (Islamic Republic of Iran and Afghanistan) and Central Asia countries are the most reported foci for CL and the major contribution of CL occur in this area (2). The CL cases in Iran during the period of 2001 to 2008 dramatically has been increased more than two-fold and also over half of 31 provinces of country have CL endemic foci (11). As reported by Center for Disease Control, the annual incidence of leishmaniasis in the country due to referring to health centers has reached about 20000 cases. While the actual cases of disease may be far more than these reports that may even reach up to five times (12).

As for the first time carried by John Snow in 1854 due to diarrheal disease detection in London, spatial modeling as a useful tool in epidemiological studies could be used. Today, Geographic Information System (GIS) in relation to the processed images from remote sensing (RS) has been used to map the geographical distributions of disease prevalence, factors affecting the transmission and control of disease, and the spatial modeling of environmental factors that have meaningful impacts on disease occurrences (9, 13). Vector control programs are associated with logistic problems, mainly due to poor understanding of vector ecology (14). Expanding inexpensive and effective models of integrated management for better control of CL is a critical target which need a thorough will, alongside epidemiology studies and understanding of the ecology of the disease (11) and the role of social, ecological and specially environmental variables for risk factors such as the vegetation and elevation (two most widespread and accessible environmental variables). The relationship between epidemiological components of leishmaniasis (incidence, prevalence, and seroprevalence) and environmental variables (vegetation, elevation, and/or other factors) can be used in GIS modeling and may be useful for secondary uses including prediction. Therefore, contributing to better planning for control activities and the establishment of early warning systems, incorporating remotely sensed information in epidemiological studies can help public health policy makers in having a better notion and comprehensive understanding to find the relationships between disease and its environmental risk factors (15).

The remotely sensed Normalized Difference Vegetation Index (NDVI) –the predominantly index which used for vegetation coverage–has found its widespread applications due to fluctuations it has along with meteorological and environmental components (14). This index together with the other remotely sensed data (11, 16) like the Digital Elevation Model (DEM), have been used extensively in monitoring some vector-borne diseases including leishmaniasis throughout the world. While many studies have surveyed the associations between the mean of NDVI and DEM with incidence or prevalence rate of a type of leishmaniasis locally, however, they do not study all variables in a vast area.

In this study, we systematically reviewed all studies regarding the incidence and/or prevalence rate of all categories of leishmaniasis investigated in Iran from 1976 to 2015. After mapping the results, using Spatial Analysis and Regression techniques, statistical association between variables have been modeled in detail.

## Materials and Methods

To identify the incidence and prevalence of leishmaniasis in endemic and nonendemic area of ZVL, ZCL, and ACL of Iran, we searched and reviewed the literature based on international database including MEDLINE, ISI, SCOPUS, and Google Scholar, and national databases such as SID, IRANDOC, MagIran, and ISC. Search protocols have been based on using the terms related to all types of leishmaniasis, fauna, vectors, reservoirs, incidence, prevalence, seroprevalence, Direct Agglutination Test (DAT), and their Persian equivalents with the geographic term “Iran”.

Overall, 503 articles published from 1976 through Jun 2015 in English, French, and Persian were founded in first search from databases. Besides, by referring to CV of famous professors, abstract seminars, thesis, non-digital papers, and the reference/citations of articles, 121 additional sources were found. After the elimination of duplicate and inappropriate articles by two separate teams, we consequently included 144 studies that clearly addressed confirmed clinical cases for human seroprevalence (DAT ≥ 1: 3200)/incidence of ZVL and active lesion prevalence/total prevalence (active lesion + scar)/incidence of ACL and ZCL in Iran. Disease data were extracted by researchers directly from body text of articles to database tables (Dbase format using the MS Excel software) containing all cities (1246 point) and villages with a population more than 500 people (12034 points), disease information, and descriptive data. Depending on the raw data presented in each article, one or more than one category of epidemiologic data was included in the main table. These numeric values (all in percent) for ACL and ZCL including active lesion prevalence, total prevalence (active lesion + scar), and incidence and for VCL including seroprevalence and incidence were directly or indirectly extracted using study population. The developed database later imported to the ArcMap ^®^ software version 9.3 (ESRI, Redlands, USA) software platform for Spatial Analyst and mapping all types of leishmaniasis. R software version 3.2.2 (17) was used for analyzing the relationship between the prevalence/incidence and continues spatial variables.

Shapefile maps for political subdivisions of provinces, counties, rural districts, and points of cities, towns, and villages with their attribute tables were obtained from National Cartographic Center, Iran. The Moderate Resolution Imaging Spectroradiometer (MODIS) onboard the Terra satellite, is one of most applied NDVI images source in literature. NDVI calculated from surface reflectance in the near-infrared ρNIR and red ρR portions of the electromagnetic spectrum, using the equation 1 (18):

1)  NDVI=(ρNIR−ρR)/ (ρNIR+ρR)

The 16d, 250m NDVI raster maps (MOD 13Q1 series, version 5) projected in Geographic Coordinate System WGS_1984, derived from MODIS Reprojection Tool (MRT) (19) for the years 2000 to 2014. Sixteen days maps unified to annual average NDVI in Spatial Analysis, then reclassified to 1–20. Altitude was based on DEM, obtained from the 100m ASTER Global Digital Elevation Model (ASTER GDEM) data center Version 2 (20). These maps then re-projected to Lambert Conformal Conic in ArcMap, and additional Raster Analysis such as reclassification, annual and 15yr average and zonal statistics were performed on them. Overall, 5km, 10km, 20km, and 50km buffer zones centered on each city/village point reported values for leishmaniasis, and their zonal Min, Max, Minority, Majority, Median and Mean statistics were calculated and exported in Dbase tables. Finally, massive joined tables were called in R, and statistical calculations were completed for each.

### Data analysis

CSV tables for any purposes derived from the main disease data table and called in R console. We chose to test the normality of data with One-sample Kolmogorov-Smirnov and Shapiro-Wilk normality test. Next, we modeled the relationships between prevalence/incidence values and the continuous independent variables. Two regression scenarios were considered. First, using simple linear regression models, a stepwise backward elimination procedure was used with the variables, finally resulting in the model contains merely the variables which associated with the outcome. Secondly, using generalized additive models -which use spline or local regression- a stepwise backward elimination procedure, was used again to reach the final model. The additive nonparametric regression model is presented in equation 2 (21):

2) $${\text{y}=\text{β}}_{0}+{\text{m}}_{1}({\text{x}}_{1})+{\text{m}}_{2}({\text{x}}_{2})+...+{\text{m}}_{\text{k}}({\text{x}}_{\text{k}})+\text{ɛ}$$

Where the partial-regression functions m _j_ are fit using a simple-regression smoother, such as local polynomial regression or smoothing splines. In nonparametric regression, in contrast, the object is to estimate the regression function directly without specifying its form explicitly. In nonparametric comparing with simple regression, the ultimate goal is to obtain the regression function directly from internal computation without any open aspect (22). The level of 5% significance for all tests was considered.

## Results

**Fig. 1.**
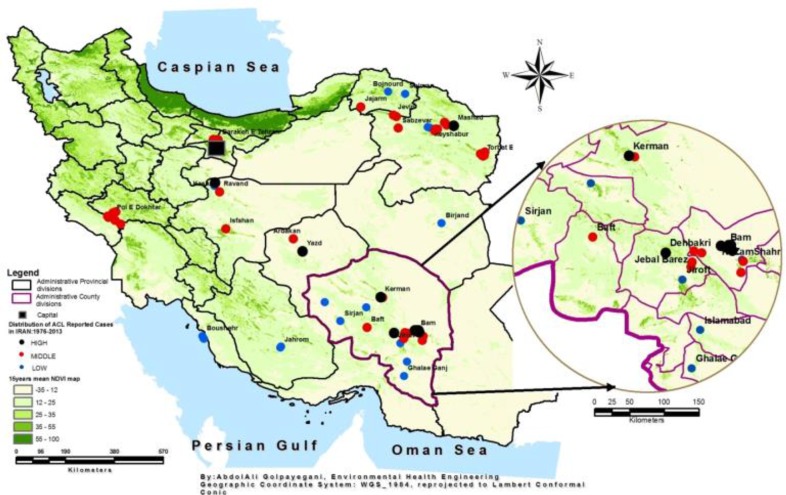
Distribution of anthroponotic cutaneous leishmaniasis city points in Iranian studies from 1976–2013 on 15yr annual average Normalized Difference Vegetation Index map. High: active lesion ≥ 2% or active lesion+scar ≥ 20% or incidence ≥ 2%; Middle: 2% > active lesion ≥ 0.5% or 20% > active lesion+scar ≥ 5% or 2 > incidence ≥ 0.5%, Low: 0.5% > active lesion or 5% > active lesion+scar or 0.5> incidence

**Fig. 2.**
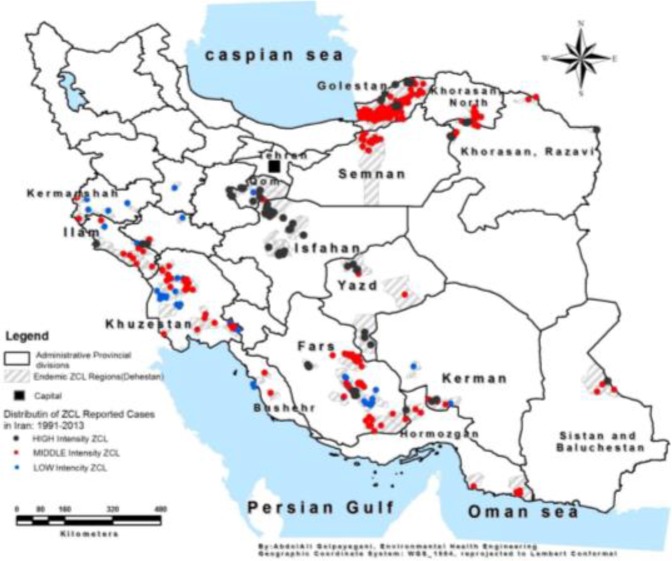
Distribution of zoonotic cutaneous leishmaniasis rural region points in Iranian studies from 1991–2013. High: active lesion ≥2% or active lesion+scar ≥20% or incidence ≥ 2%, Middle: 2% > active lesion ≥ 0.5% or 20% > active lesion+scar ≥ 5% or 2> incidence ≥ 0.5%, Low: 0.5% > active lesion or 5% > active lesion+scar or 0.5 > incidence

**Fig. 3.**
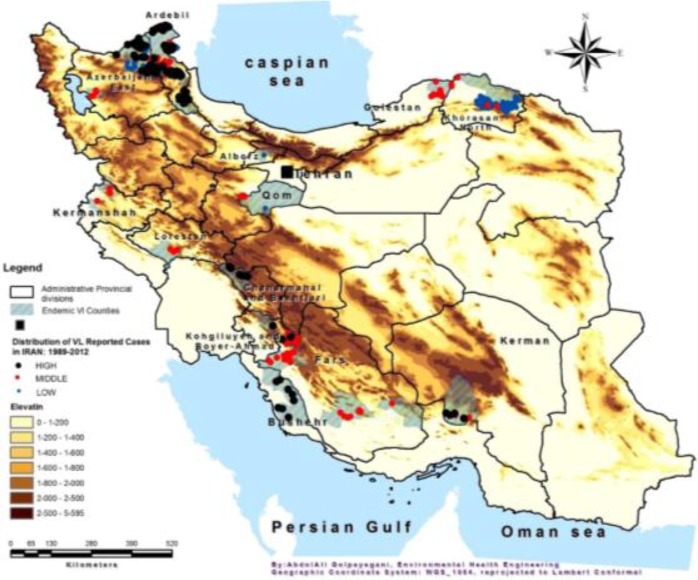
Distribution of zoonotic visceral leishmaniasis county region points in Iranian studies from 1989–2012 on Digital Elevation Model map. High: DAT ≥ 2% or incidence ≥ 1%, Middle: 2% > DAT ≥ 0.5% or 1 > incidence ≥ 0.1%, Low: 0.5% > DAT or 0.1 > incidence

We found 144 studies that directly informed the active lesion/scar prevalence and/or incidence of leishmaniasis in Iran from 1976–2015 (Table 1). These data considered as a base for construction of 89 ACL points including 15, 19, and 71 urban/rural points for active lesion, total prevalence (active lesion + scar), and incidence respectively. The same way, data for 420 ZCL points included 67, 68, and 362 points. Cases for 355 ZVL points for the seroprevalence and incidence were 283 and 82 respectively. Figures 1, 2, and 3 show the maps of three types of leishmaniasis in Iran separately. The DEM map and 15yr average of NDVI map also attached on VL and ACL maps respectively. Rating in the categories are High, Middle and Low for prevalence data and obtained based on expert consensus.

**Table 1.** Literatures published for prevalence of leishmaniasis, divided by leishmaniasis types and provinces of Iran

**Province**	**ACL references**	**ZCL references**	**ZVL references**
**Alborz**			Fagih-Nayini et al. 2002(23)
**Ardabil**			Mazloumi Gavgani et al. 2002(24), Soleimanzadeh et al. 1993(25), Mohebali et al. 2010(26), Edrissian 1996(27), Mohebali et al. 2011(28), Mahami et al. 2006(29), Mohammadi-Kheyrabadi et al. 2004(30), Nadim et al. 1998(31), Arshi et al. 2002(32), Mazloumi Gavgani et al. 2011(33)
**Azerbaijan, East**			Mazloumi Gavgani et al. 2002(24), Mohebali et al. 2011(28), Mirsamadi et al. 2003(34), Mazloumi Gavgani et al. 2011(33)
**Azerbaijan, West**			
**Bushehr**	Yaghoobi-Ershadi et al. 2013(35)	Yaghoobi-Ershadi et al. 2013(35), Hamzavi et al. 2000(36)	Mohebali et al. 2001(37)
**Chahar Mahaal and Bakhtiari**			
**Fars**	Ghatee et al. 2013(38)	Razmjou et al. 2009(39), Ghatee et al. 2013(38), Rassi et al. 2004(40), Fakoorziba et al. 2011(41), Davami et al. 2010(42), Sharafi et al. 2013(43), Noorpisheh et al. 2013(44), Entezari and Eskandari 2014(45)	Fakhar et al. 2008(46), Edrissian et al. 1993(47), Sahabi et al. 1992(48), Fakhar et al. 2006(49)
**Gilan**			
**Golestan**		Mollalo et al. 2015(50), Rahbarian et al. 2009(51), Sofizadeh et al. 2012(52), Baghaei et al. 2012(53), Mesgarian et al. 2010(54)	(Fakhar et al. 2014)
**Hamadan**		Hanafi Bojd et al. 2006(55), Nazari 2012(56)	
**Hormozgān**		Azizi et al. 2012(57)	
**Ilam**		Asgari Nezhad et al. 2012(58), Shazad et al. 2005(59), Bahrami et al. 2011(60), Yazdanpanah and Rostamianpur 2013(61), Roghani et al. 2012(62)	
**Isfahan**	Shiee et al. 2012(63), Zahraei-Ramazani et al. 2007(64), Doroodgar et al. 2012(65)	Talari et al. 2006(66), Yaghoobi-Ershadi et al. 2001(67), Mohammadian et al. 1999(68), Yaghoobi-Ershadi and Javadian 1995(69), Yaghoobi-Ershadi et al. 2006(70), Nilforoushzadeh et al. 2002(71), Doroodgar et al. 2009(72), Ahmadi et al. 2013(73), Ebadi and Hejazi 2003(74), Yaghoobi-Ershadi et al. 1999(75), Doroudgar et al. 1996(76)	
**Kerman**	Nadim and Aflatoonian 1995(77), Sharifi et al. 2011(78), Sharifi et al. 2015(3), Yaghoobi-Ershadi 1977(79), Mirzaei et al. 2012(80), Aflatoonian et al. 2014(81), Sharifi et al. 1998(82), Sharifi et al. 2011(83), Ghatee et al. 2013(38), Aflatoonian et al. 2013(84), Nadim et al. 1995(85), Sharifi et al. 2015(86), Aflatoonian and Sharifi 2011(87), Aflatoonian and Sharifi 2010(88), Mirzadeh et al. 2008(89), Aflatoonian et al. 2014(90), Aflatoonian and Sharifi 2007(91)	Sharifi et al. 2015(3), Akhavan et al. 2007(92), Aghaei-Afshar et al. 2013(93), Khosravi et al. 2013(94), Sharifi et al. 2008(95)	Mahmoudvand et al. 2011(96), Barati et al. 2008(97)
**Kermanshah**		Hamzavi and Khademi 2015(98), Nazari et al. 2012(99), Hamzavi and Khademi 2013(100)	Hamzavi et al. 2012(101)
**Khorasan, North**	Alavinia et al. 2009(102)	Alavinia et al. 2009(102)	Torabi et al. 2007(103)
**Khorasan, Razavi**	Sattar Pagheh et al. 2013(104), Moosa-Kazemi et al. 2007(105), Saadabadi et al. 2013(106), Hassanpour et al. 2014(107), Hoseini Farash et al. 2011(108), Khajedaluee et al. 2014(109), Mohajery et al. 2008(110)	Yaghoobi-Ershadi et al. 2003(111), Hassanpour et al. 2014(107), Khajedaluee et al. 2014(109), Karimi Zarchi et al. 2004(112), Sahabi et al. 1999(113), Akbari et al. 2014(114)	
**Khorasan, South**		Karamian et al. 2013(115)	
**Khuzestan**		Kassiri et al. 2012(116), Kassiri et al. 2012(117), Kassiri et al. 2014(118), Kassiri et al. 2014(119), Vazirianzadeh et al. 2014(120), Kassiri et al. 2013(121), Spotin et al. 2014(122), Ghasemian et al. 2011(123), Kassiri et al. 2013(124), Nejati et al. 2014(125), Behbahani et al. 2012(126), Kassiri et al. 2011(127)	
**Kohgiluyeh and Boyer-Ahmad**			Sarkari et al. 2010(128), Sarkari et al. 2007(129)
**Kurdistan**			
**Lorestan**	Kheirandish et al. 2013(130)	Amraee et al. 2013(131), Ahmadi et al. 2013(132), Kheirandish et al. 2013(130), Chegeni-Sharafi et al. 2011(133)	Chegeni-Sharafi et al. 2005(134)
**Markazi**			
**Mazandaran**			
**Qazvin**			
**Qom**		Rostami et al. 2013(135), Rassi et al. 2013(12), Akhavan et al. 2003(136), Saghafipour et al. 2013(137), Saghafipour et al. 2012(138)	(Rakhshanpour et al. 2014), (Fakhar et al. 2004)
**Semnan**		Mohammadi Azni et al. 2010(139), Mohammadi Azni et al. 2011(140), Mohammadi Azni et al. 2010(141), Rafati et al. 2007(142)	
**Sistan and Baluchestan**		Fazaeli et al. 2008(143), Fazaeli et al. 2009(144), Fouladi et al. 2007(145)	
**Tehran**	Salehzadeh and Seyedi Rashti 1996(146), Hossein Abadi 2004(147)		
**Yazd**	Yaghoobi-Ershadi et al. 2002(148)	Yaghoobi-Ershadi et al. 2004(149), Jaafary et al. 2007(150), Mozafari and Bakhshi Zadeh 2011(151), Hanafi Bojd et al. 2003(152), Yaghoobi-Ershadi et al. 2008(153)	
**Zanjan Multy-provinces**			Mohebali et al. 2006(6)

**Fig. 4.**
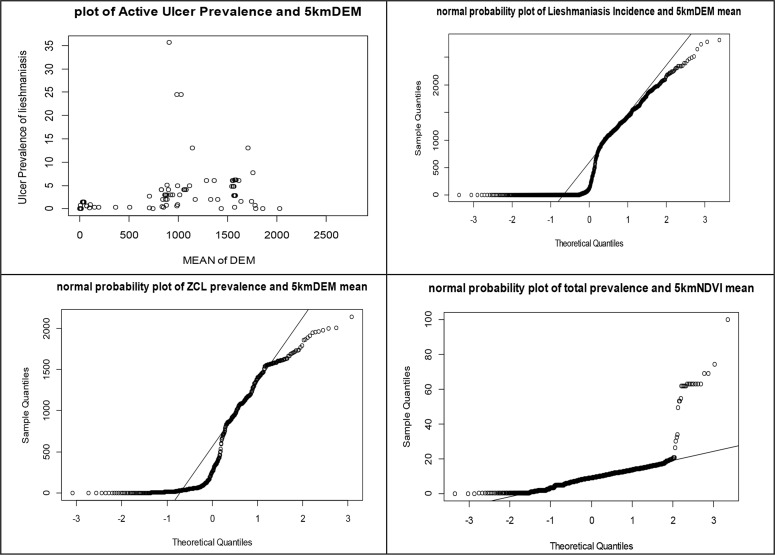
Typical plots and normality plots of independent and dependent variables

Scatter diagrams and normality plot of each independent variable and the disease incidence/prevalence were created and visually inspected before running regression. Some of these plots traced in Fig. 4. Normal probability of data in our variables has been questioned, then, the normality of data statistically tested. Therefore, using both One-sample Kolmogorov-Smirnov and Shapiro-Wilk normality test, the data are without normality. However, both linear and nonparametric regression fitted on data.

**Fig. 5.**
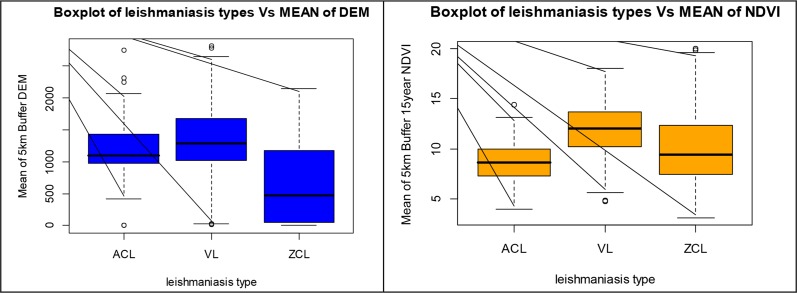
Boxplots for compares of means of 5km-buffer Digital Elevation Model and Normalized Difference Vegetation Index ((in the range of 1–20)) for anthroponotic cutaneous leishmaniasis, zoonotic cutaneous leishmaniasis, and zoonotic visceral leishmaniasis in Iran for study period (1976–2013)

To compare of the means of main independent variables (DEM and NDVI) versus types of leishmaniasis, homogeneity of variances rejected by Fligner-Killeen test, but due to nonparametric Kruskal-Wallis test, we result in significant difference between means of variables. Regard to boxplots for comparison of means in Fig. 5, and nonparametric analysis of variances in Table 2, tendency of ZVL to presence in high elevation and high vegetation is more than ACL and ZCL. Similarly, we compare the means of types of leishmaniasis occurrences and epidemiological variables of disease in Table 3.

**Table 2.** Nonparametric analysis of variances between types of zoonotic, anthroponotic cutaneous, and zoonotic visceral leishmaniasis and environmental variables in Iran (P< 0.05), buffer = 5km

	**DEM_MEAN**	**DEM_VARIANCE**	NDVI_MEAN^*^	**NDVI_VARIANCE**
**ACL**	1216	213046	8.57	3.93
**ZCL**	666	385752	10.12	12.16
**ZVL**	1296	332153	11.93	6.28

* (The range is 1–20 for NDVI)

**Table 3.** Nonparametric Analysis of variances between types of zoonotic, anthroponotic cutaneous, and zoonotic visceral leishmaniasis and epidemiological variables of disease in Iran (P< 0.05)

	**MEAN**			**VARIANCE**		

	**ACL (%)**	**ZCL (%)**	**ZVL (%)**	**ACL**	**ZCL**	**ZVL**
**Active lesion prevalence**	1.48	4.42	Na^*^	3.47	37.97	Na
**Total prevalence**	11.88	25.02	2.69	125.38	622.39	3.73
**Incidence**	0.76	1.04	1.01	2.62	8.47	1.78

* Na: Not available

The regression models were evaluated using R software packages. Tables 4 and 5 generally descript data structure and assessment of the effect of, or relationship between, explanatory variables on the response by using multiple linear regression models.

**Table 4.** Multiple linear regression models for types of zoonotic, anthroponotic cutaneous, and zoonotic visceral leishmaniasis and Digital Elevation Model subcategory (buffer distance around the cities or villages)

	**DEM 5km_total**	**DEM 5km_ACL**	**DEM 5km_ZVL**	**DEM 5km_ZCL**	**Buffer types**	**Buffer types_ACL**	**Buffer types_ZVL**	**Buffer types_ZCL**
**Active lesion prevalence**	∼MEAN+MAX AR^*^ : 0.1004	∼MEAN+MIN AR: 0.284	Na^***^	∼MEAN AR: 0.077	∼MEAN20km AR: 0.035	ns	ns	∼MEAN5km AR: 0.077
**Total prevalence**	∼MAX AR: 0.1028	∼MEAN AR:0.1649	∼MAX AR:0.0484	∼MEAN+MAX AR: 0.234	∼MEAN20km AR: 0.1178	ns	∼MEAN50km AR: 0.029	∼MEAN20km AR: 0.25
**Incidence**	∼MEAN+MAX AR: 0.029	Ns^**^	∼MIN AR:0.2954	∼MAX+MAJORITY AR: 0.077	∼MEAN5km AR: 0.011	ns	∼MEAN20km AR: 0.21	∼MEAN5 AR: 0.031

* AR: Adjusted R-squared

** Ns: Not significant

*** Na: Not available

**Table 5.** Multiple linear regression models for disease mode and Normalized Difference Vegetation Index subcategory (buffer distance around the cities or villages)

	**NDVI 5km_total**	**NDVI 5km_ACL**	**NDVI 5km_ZVL**	**NDVI 5km_ZCL**	**Buffer types**
**Active lesion prevalence**	∼MEAN+MAJORITY AR^*^ : 0.0338	^**^ Ns	^***^ Na	Ns	∼MAX50km AR: 0.093
**Total prevalence**	∼MIN AR: 0.012	Ns	∼MEAN AR:0.39	∼ MIN + MINORITY AR: 0.3479	∼ MIN5km AR: 0.012
**Incidence**	∼MEAN AR: 0.0085	∼ MINORITY AR: 0.042	Ns	∼MEDIAN AR: 0.0259	∼MEAN20km AR: 0.007

* AR: Adjusted R-squared

** Ns: Not significant

*** Na: Not available

To determine the best final fitted models for each group of data, based on P-value, adjusted R-squared, and AIC (Akaike's 'An Information Criterion'), using stepwise backward simple linear regression, following model produced: (although second order or interaction models had a little more R-squared, due to the complexity were excluded).

3) $$\begin{array}{l} \text{Total}\;\text{prevalence}=\;12.14-0.014\;({\text{MEAN\$DEM}}_{20})+0.005\;({\text{MEAN\$DEM}}_{50})-1.64\;({\text{MIN\$NDVI}}_{20})+1.8({\text{MEAN\$NDVI}}_{20})\\ \text{Multiple}\;\text{R}-\text{squared}:\;0.2111,\;\text{Adjusted}\;\text{R}-\text{squared}:\;0.198\\ \text{F}-\text{statistic}:\;16.06\;\text{on}\;4\;\text{and}\;240\;\text{DF},\;\text{P}:1.151{\text{e}}^{-11} \end{array}$$

**Fig. 6.**
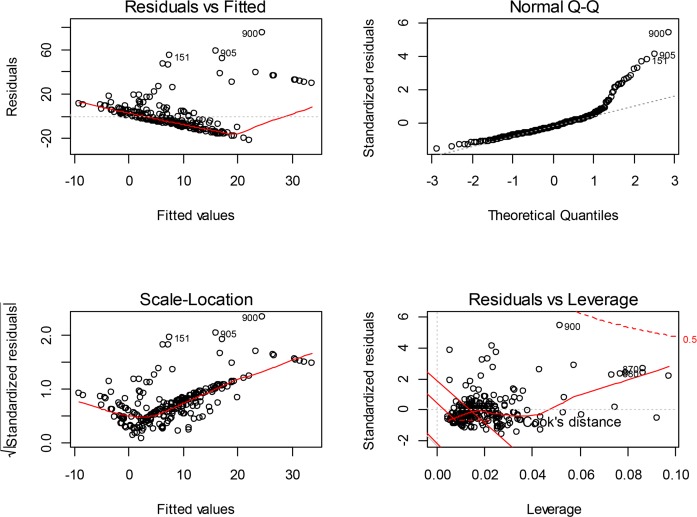
Plots of final linear regression model for leishmaniasis and environmental variables (Digital Elevation Model and Normalized Difference Vegetation Index)

Important plots of main model presented in Fig. 6. Regarding weak results of linear regression (R-squared located near the zero), eventually, Additive Nonparametric Regression analysis, using GAM ( ^1^ Generalized Additive Model) command in library “mgcv”, applied to find best parameters and buffers for modeling variables of this study. Here two models were fitted: first with the highest percentage of predictability by equation 4 , second with the convenience practical application (same parameter mean of variables in unique buffer 10km) which came in equation 5 .

4) $$\text{Total}\;\text{prevalence}=\;7.69+\;\text{s}({\text{MEAN\$DEM}}_{10})+\text{s}({\text{MAX\$NDVI}}_{50})\;\text{R}-\text{sq}.\;(\text{adj})=0.524\;\text{Deviance}\;\text{explained}=54.5\%$$

5) $$\text{Total}\;\text{prevalence}=\;7.42+\;\text{s}({\text{MEAN\$DEM}}_{10})+\text{s}({\text{MEAN\$NDVI}}_{10})\;\text{R}-\text{sq}.\;(\text{adj})=0.371\;\text{Deviance}\;\text{explained}=39.6\%$$

**Fig. 7.**
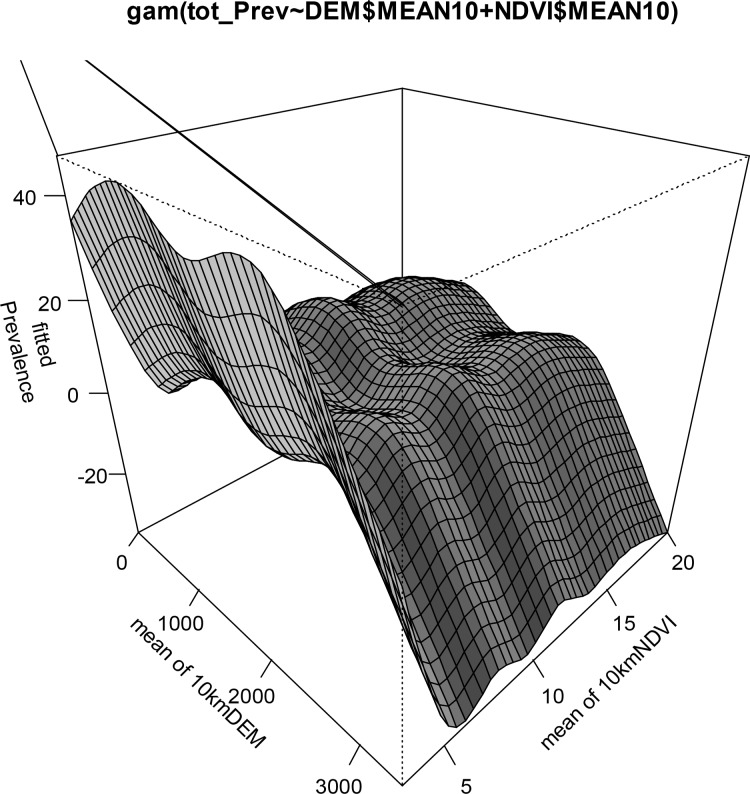
Fitted surface for the additive nonparametric regression of total prevalence of leishmaniasis on mean of 10km buffer for Digital Elevation Model and Normalized Difference Vegetation Index

**Fig. 8.**
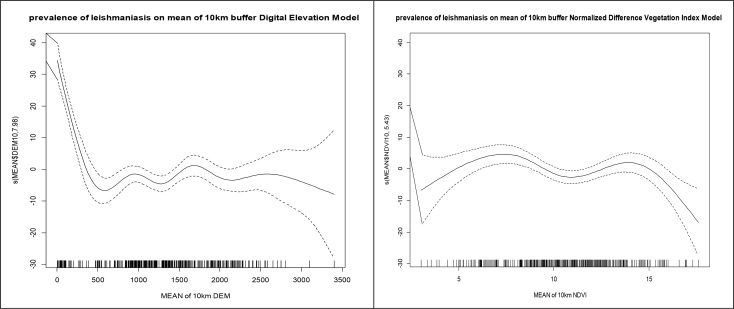
Partial-regression functions for the additive regression of total prevalence of leishmaniasis on mean of 10km buffer Digital Elevation Model and Normalized Difference Vegetation Index. The broken lines give point-wise 95-percent confidence envelopes around the fit

The “s” function, used in specifying the model formula, indicates that each term is to be fitted with a smoothing spline. The surface that fitted on the model and smoothing splines have been shown in Fig. 7 and 8. In equation 5 , the 5.43 parameters are used for the MEAN$NDVI _10_ (mean of 10km buffer for NDVI) term, and 7.98 for the MEAN$DEM _10_ term, the degrees of freedom for the model is the sum of these plus 1 for the regression constant.

## Discussion

Maps based on the actual data for distribution of all leishmaniasis types in Iran, with identifying the detailed variables of environmental factors affecting the disease distribution, are the most important findings of this study. Due to the limited access to official leishmaniasis data of Center for Disease Control, integration all data from both CL types and rural and urban foci in the heart of the county official reports (lack of separation species), tens of kilometers distance between some foci and center of counties often identified as disease points (lack of spatial resolution), unknown types of CL in most of official data, and passive screening in Public Health Centers, we decided to use the real data available in the literatures with a detailed focus on disease points. Many of scientific epidemiological studies predominantly focus on foci of diseases and have very valuable and verified information about all aspects of the disease, such as prevalence, incidence, vectors, reservoirs, and control of disease data. Maps that contain all of the scientific information investigate by researchers of a country over several decades could well represent the disease situation in that country. Although the nature of the leishmaniasis itself was not the aim of our study, however, distribution of disease to the breakdown mapping can present more accurate view of disease for researchers.

Though vegetation statistically has a significant relationship with the prevalence of vector-borne diseases, this study (especially for the simple linear regression models), with a weak confirmation, implicitly point to a complex and indirect relationship. Therefore, more detailed studies and comprehensive investigations would be needed to prove the theorem. A study on effect of minimum, maximum and mean NDVI on the distribution of the VL vector in district of Bihar, India showed that max and mean variables with an R ^2^ about 0.6 strongly associated with the disease (14). Werneck et al. with a mention to the NDVI, emphasized on the impact of multilevel variable modeling on the development of leishmaniasis in Brazil (154). Study of environmental factors influencing the distribution of vectors involved in transmission of disease in north-eastern Italy revealed that areas with high winter NDVI may be related to the survival of the larvae in moist soil (155). According to the results of relationship between CL and NDVI in northeast province of Iran, Golestan, arid and semi-arid regions with low vegetation are the most involved area for CL occurrence (11). In our study, as expected due to their rural and nomadic natures of ZCL and ZVL in Iran, the values for NDVI index increase for ACL, ZCL, and ZVL respectively (Table 2). There is not much variation among them, and all three types are almost inclined to intermediate vegetation (neither too sparse nor too dense). The distinction in the result of numerous studies would be aroused from differences between climate factors, socio-economic variables, instrumental confounders and differences in the interpretation of the results.

Incidence of various types of leishmaniasis in different heights may result from the diversity in the reservoirs and vectors of the disease. Whereas ACL and ZVL had an interest for presence in high altitude areas, ZCL have been distributed in lower altitude (Table 2). This result for ZVL has obtained also in Ilam Province spatial modeling, and elevation had a negative impact on CL prevalence (156). Multivariate analysis in a VL study in eastern Sudan found the elevation as important variables, while the primary analysis did not show any correlation with disease incidence (157). However, while DEM can use as a good variable for topographic purposes, it may do not has most appropriate function as elevation in all regions (155).

The role of environmental factors in the development of disease vectors is obvious. While the CL is the main vector-borne disease in Iran, about 80% of cases are zoonotic and spread in 17 out of 31 provinces (158). Some studies have been modeled the environmental factors for vectors and reservoirs of leishmaniasis. Altitude and land cover was found to be from most important factors for suitability of niches for sand flies. Areas with 990m were found and 1235m average altitude were related to probability of presence of ZCL and ACL vectors respectively (159). Niche modeling of main reservoir hosts in Iran showed that among topography variables, slope has the most contribution in forecasting risk of disease (160). A predictive degree-day model was used for development time, population dynamics and activities of VL vector sandflies in field in northwest Iran (161). Among climate factors, minimum of temperature, mean of humidity, and rainfall had the most impact on ZVL distribution in Golestan, north-east of Iran (162). None of these studies include NDVI as a variable.

The vegetation coverage has been discussed as a risk factor for VL and CL types in previous studies (163, 164). The NDVI has not considered for intrinsic specifications itself but also it covers some other important environmental and ecological variables such as soil types and moisture, humidity, slope degree and even elevation of its ambient land (16). The majority of studies, evaluated the presence/absence of vegetation or have used a cut-off value point for NDVI, but less attention has been paid to the sheer numerical modeling. Linear regression results in this study show that none of subcategories of independent variables have a favorable associated with DEM and NDVI and do not fit on the linear model very well (low R-squared). Only some of models when focused on leishmaniasis types, the R-squared reach to 0.2–0.4. This fact could be predicted from plots associated with variables (Fig. 4). Despite the lack of goodness of fitness of simple linear models, Additive Nonparametric Regression analysis presents a better prediction model for data of this study. These models do not output real intercepts for variables, but using their internal formulas, they are able to provide predictive models for input data. As a nonparametric regression attributes, no obvious estimates were seen for parameters (21), and then to figure out regression results, regards to necessitate of using fitted regression graphics, we used this method as shown in Fig. 7. Data frame used to find predicted values on the regression surface, fitted to data of model to drawn this figure.

For more clarification, functions which explained previously that present in inherent content of the additive regression, have been shown in partial regression of our model in Fig. 8.

## Conclusion

Although the results of modeling and predictive power of the models in this study was not great but was somewhat satisfied. Modeling environmental factors which affecting ecological disease, need attention to the role of all these factors together. Moreover, integration the scenarios such as finding hotspots in GIS, using statistical logistic regression, more specific factors of diseases such as vector and reservoir species, can be used in modeling the relationships, more meaningful and more clear. However, a good model needs to consider all the factors involved the prevalence and incidence of a disease.
